# Assessing climate change impacts on Pacific salmon and trout using bioenergetics and spatiotemporal explicit river temperature predictions under varying riparian conditions

**DOI:** 10.1371/journal.pone.0266871

**Published:** 2022-05-20

**Authors:** Andrew R. Spanjer, Andrew S. Gendaszek, Elyse J. Wulfkuhle, Robert W. Black, Kristin L. Jaeger

**Affiliations:** 1 U.S. Geological Survey, Washington Water Science Center, Tacoma, WA, United States of America; 2 Department of Natural Resources, Quinault Indian Nation, Taholah, WA, United States of America; Centro de Investigacion Cientifica y de Educacion Superior de Ensenada, MEXICO

## Abstract

Pacific salmon and trout populations are affected by timber harvest, the removal and alteration of riparian vegetation, and the resulting physical changes to water quality, temperature, and associated delivery of high-quality terrestrial prey. Juvenile salmon and trout growth, a key predictor of survival, is poorly understood in the context of current and future (climate-change mediated) conditions, with resource managers needing information on how land use will impact future river conditions for these commercially and culturally important species. We used the Heat Source water temperature modeling framework to develop a spatiotemporal model to assess how riparian canopy and vegetation preservation and addition could influence river temperatures under future climate predictions in a coastal river fed by a moraine-dammed lake: the Quinault River in Washington State. The model predicted higher water temperatures under future carbon emission projections, representative concentration pathway (RCP) 4.5 and 8.5, with varying magnitude based on different riparian vegetation scenarios. We used the daily average temperature output from these scenarios to predict potential juvenile fish growth using the Wisconsin bioenergetics model. A combination of riparian vegetation removal and continued high carbon emissions resulted in a predicted seven-day average daily maximum temperature (7DADM) increase of 1.7°C in the lower river by 2080; increases in riparian shading mitigate this 7DADM increase to only 0.9°C. Under the current thermal regime, bioenergetics modeling predicts juvenile fish lose weight in the lower river; this loss of potential growth worsens by an average of 20–83% in the lower river by 2080, increasing with the loss of riparian shading. This study assess the impact of riparian vegetation management on future thermal habitat for Pacific salmon and trout under warming climates and provide a useful spatially explicit modeling framework that managers can use to make decisions regarding riparian vegetation management and its mechanistic impact to water temperature and rearing juvenile fish.

## Introduction

Water temperature is a vital component of riverine environments that controls the location, structure, and function of freshwater habitat. Climate change, resulting in increased air temperature, is predicted to increase river and stream temperatures in many places [1–4]. These increasing river temperatures directly impact freshwater fish by creating barriers to migration [5–7], controlling growth and metabolism [8,9], and influencing water quality parameters [10]. Pacific salmon and trout (*Oncorhynchus spp*.*)* are particularly vulnerable to temperature extremes [11]. Healthy populations of Pacific salmon and trout species are valued for their cultural [12,13], subsistence, economic, and recreational significance resulting in hundreds of millions of dollars spent annually in the Pacific Northwest of the United States toward habitat restoration [14]. Understanding the complex integration of physical controls on river temperature and its impact on these fish’s ecological success can help define and guide restoration and conservation efforts. Modeling and prediction tools are needed to better integrate these complex physical controls on water temperature at scales relevant to environmental managers deciding where to invest limited resources to improve habitats and adaptively plan for a changing climate.

Process-based water temperature modeling is an ongoing area of research [15,16] capable of integrating and predicting the impact that weather, riparian vegetation, and flow changes have on river temperature [17–20]. Application of process-based temperature models to specific rivers can be more resource-intensive than their statistical counterparts [16,21]. Yet, these types of models are better suited to address the efficacy of management actions to offset future temperatures, integrating both future climate conditions and physical changes at the reach scale. Additionally, the output from these models is well suited for developing temperature inputs for fish bioenergetic models. Bioenergetics models use an energy balance equation to describe the fundamental physiological processes associated with fish metabolism, waste, consumption, and growth [22–25]. They are extensively used and well corroborated for predicting growth potential in Pacific salmon habitats [8,26,27]. Juvenile salmon and trout growth is an essential indicator of fish health and habitat quality linking to long-term survival and run size [28,29] through size-selective mortality during early freshwater and marine life stages [8]. Because temperature has nonlinear effects on fish consumption and metabolism [30,31], summertime water temperatures can exceed physiological thresholds resulting in stress, weight loss, and potential death. As such, summertime river temperatures are a common stressor for many populations of Pacific salmon and trout [11].

The lower Quinault River provides critical habitat for six species of anadromous Pacific salmon and trout (*Oncorhynchus spp*.). Chinook (*O*. *tshawytscha*), chum (*O*. *keta*), coho (*O*. *kisutch*), steelhead (*O*. *mykiss*) and cutthroat (*O*. *Clarkii)* use the lower river for migration, spawning, and rearing, while sockeye (*O*. *nerka*) use the channel for migration to and from the lake [32,33]. The river supports tribal and recreational salmon fisheries. One way that timber harvesting throughout the basin can degrade the physical river habitat is through the removal and alteration of riparian vegetation. Healthy populations of important salmon stocks are influenced by these physical changes and the resulting impact on water quality. Potential for salmonid growth throughout the lower river basin are poorly understood under current and future climate and habitat conditions.

The lower Quinault River’s temperature regime is influenced by the water discharge from the naturally occurring moraine-dammed lake, Lake Quinault. The lake’s solar warmed surface water likely leads to elevated downstream water temperature during the summer [34,35]. Temperature tolerance for Pacific salmon and trout depends on species and life stage. Freshwater thermal limits for spawning, incubation, and emergence are generally around 13°C, temperatures limiting juvenile rearing and adult migration are above 16°C, and direct mortality occurs above 24°C [36]. In 2016, Jaeger and others [37] documented high water temperatures within the lower Quinault River with daily minimums exceeding 16°C and summer maximum daily water temperatures ranging from 20° to 25° C, well exceeding EPA thermal thresholds for salmon. Their study additionally surveyed the lower Quinault river for discrete cold-water refuge within the thermal tolerances for salmon [37]. Cold-water refuge locations were related with river-side shading from solar radiation, suggesting that riparian vegetation preservation and addition could influence river temperatures throughout the reach. An additional assessment was needed to inform how riparian management throughout the lower Quinault River basin (from the lake outlet to the estuary) would influence temperature to preserve and improve the growth habitat for fish.

We coupled a process-based water-temperature model to a juvenile salmon and trout growth model to investigate the potential impact of climate change and riparian management on river temperatures and thermal habitat for juvenile Pacific salmon and trout species, mainly Chinook salmon. By coupling these models, we predict how the magnitude of shading from riparian vegetation impacts fish growth conditions in the lower Quinault River, WA, USA. We further consider future climate conditions using a locally downscaled global climate model under two predicted atmospheric carbon concentration scenarios, representative concentration pathway (RCP) 4.5 and 8.5 [38]. Output from these models were analyzed to meet our study’s specific objectives: 1) predict riparian shading effects on fish growth; 2) estimate how future climates affect fish growth under the two RCP climate projections; 3) investigate the interactive effects of shading and future climates; and 4) spatially identify life-history specific temperature threshold exceedances for Pacific salmon and trout species.

## Methods

### Study area

**Fig 1:**
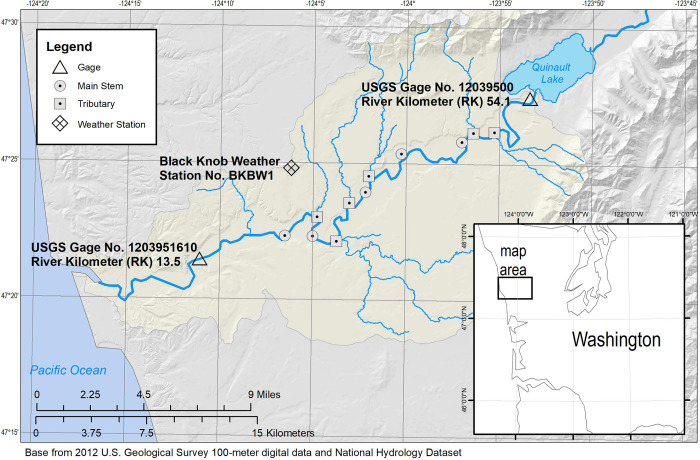
Map of temperature monitoring locations throughout the modeled reach of the Quinault River, Washington. Temperature loggers were deployed at each location, either on the main stem or on tributaries flowing into the Quinault River.

The Quinault River flows from its headwaters of the Olympic Mountains within the Olympic National Park to the Pacific Ocean, draining 1,134 km^2^ (Fig 1). Lake Quinault, a moraine-dammed lake, separates the lower and upper sections of the river at approximately river kilometer (RK) 54.1 and RK 56.6 [39], respectively. For reference, RK 0.0 is located at the mouth of the Quinault where it enters the Pacific Ocean. The lake attenuates peak-flow magnitude in the lower Quinault River and restricts downstream delivery of coarse sediment (sand and coarser) and large wood from the upper Quinault River. Also, the lake affects water temperature in the lower Quinault River, and relatively high summer water temperatures, as compared to other nearby rivers, are common throughout the lower river [37]. The lower Quinault River sub-watershed is located entirely within Quinault Indian Nation land. The floodplain is primarily forested but has been subject to timber harvest activities since the late 1920s [39]. Two U.S. Geological Survey (USGS) stream gages are currently in operation in the lower Quinault River; one at the outlet of Lake Quinault (USGS stream gage 12039500) has been in operation since 1916, and a more recent gage at RK 13.5 (USGS stream gage 1203951610) (Fig 1) was installed in 2017.

**Fig 2:**
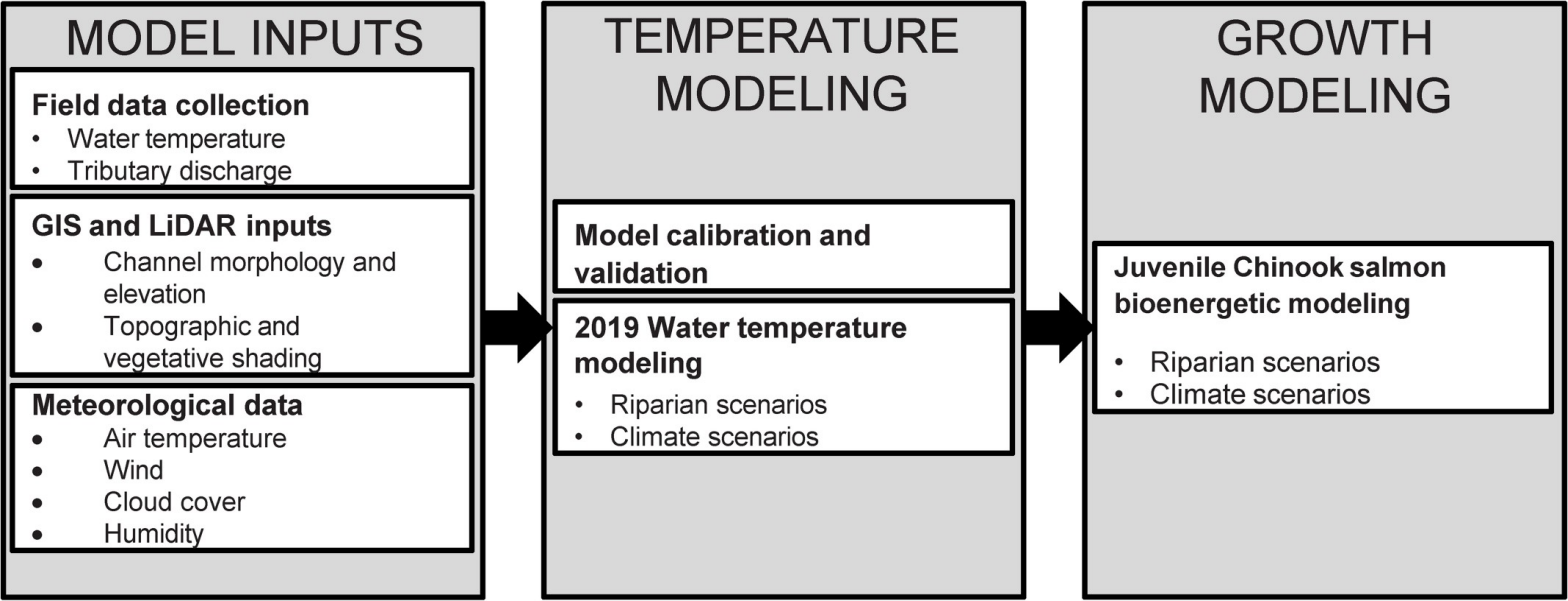
Modeling framework. Conceptual diagram of modeling of inputs needed to run, parameterize, and link temperature and growth modeling.

To understand how riparian conditions and climate change impact Pacific salmon and trout growth in the lower Quinault River (Fig 1), we linked a water-temperature model with a bioenergetics model to predict how river temperature in the lower watershed affected Chinook salmon growth potential following the steps outlined in Fig 2. Modeling focused on juvenile Chinook salmon, as they are likely to use the lower river for rearing in summer months when temperatures are warm and because their physical response to temperature is similar to other anadromous salmonid species (see discussion for details). To run these models, we measured and estimated Lake Quinault outlet and inflowing tributary thermal regimes, discharge, meteorological, morphological, and landcover data throughout the lower Quinault River basin during the summer growing period for Chinook salmon. These data were used as inputs to Heat Source [40], a process-based water-temperature model, to predict hourly river temperature at every 500 meters along a roughly 41-kilometer (km) reach of the lower Quinault River near Angel Park, Washington. The modeled reach extent was delineated from the location the two permanent USGS gages mentioned above (Fig 1). This distance covers most of the river between the lake and estuary near Taholah, Washington. Predicted hourly temperatures under different shading and climate conditions were used as input for the Wisconsin Fish Bioenergetics [41] model that predicts fish growth at an hourly time step with inputs of fish size, consumption rate, daily water temperature, prey energy, and predatory energy density. Bioenergetics modeling and statistical analysis were conducted using R-statistical software [42]. Visualization and graphing of data used R base and ggplot2 [43] packages, ArcMap 10.6, and ArcMap pro [44].

### Heat source water temperature model

Field measured water temperature and tributary discharge were collected for input into the temperature model (Fig 2). Water temperature (°C) was collected during the summers of 2018 and 2019 at 13 locations (Fig 1 and supplemental information, Table S1 in S1 File), recorded at hourly intervals. Five water-temperature measurement sites were in the Quinault River’s main stem, and six were at tributary locations. Limited road access exists along the lower river, and deployment periods and locations were restricted by raft-only access. The two USGS gaging stations supplied the upper and lower modeling boundary temperature (only 2018 data were used from the upstream boundary gage 12039500). The temperature at all other locations, including the upper boundary in 2019, was supplied by water temperature loggers (HOBO® Water Temp Pro v2 or TidbiT v2). At each location where a temperature logger was placed, quality assurance measures were taken, including cross-section measurements made at each location to assure uniform mixing across the river, loggers were checked against a NIST certified field meter, and, in 2019, temperature logger accuracy was checked in a cold and warm water bath following USGS guidelines [45]. Field temperature data generated during this study with detailed quality control descriptions are available via a USGS Sciencebase data release [46] available at https://doi.org/10.5066/P9GSX4QE, and discharge measurements are available via the National Water Information System (NWIS). [47]: https://www.waterdata.usgs.gov/nwis.

The Heat Source model relies on measures and estimates of boundary conditions and tributary inflow. The USGS Lake Quinault outlet gage (12039500) provided data for the upstream boundary at the needed hourly time scale. Discharge measures at tributary locations (Fig 1 and supplemental information Table S1 in S1 File, discharge data available from NWIS) were made with a handheld acoustic Doppler velocimeter (Sontek® FlowTracker) during baseflow conditions in 2018. The measured tributary discharge values were compared to upstream gaged flow to calculate tributary flow as a percent of upstream gaged flow. This percentage was held constant over the modeling period, and the tributary inflows were estimated on an hourly timestep. Given the low variability in summer flows on the lower Quinault River, the simple percentage relationship between tributaries and the main stem is a reasonable estimation of flow.

River channel attributes, riparian vegetation, and topographic elevation were input into the model to determine radiative shading throughout the reach. TTools [48], a python-based add-in for ArcGIS Desktop, was used to determine river channel attributes and near river effective shading from vegetation and topography along the 41-kilometer reach from RK 54.1 down to RK 13.5. We first delineated the river channel and streambanks using orthoimages and GPS tracks from rafting the river in 2018. Additionally, raster elevation layers of regional topography, localized ground elevation, and near-river vegetation height were created or acquired as described below.

Publicly available light detection and ranging (LiDAR) data were processed to construct a raster layer of vegetation heights and localized ground elevation along the lower reach. Two LiDAR datasets, completed for the Quinault River basin in 2011 and 2009, were downloaded from the Puget Sound LiDAR Consortium [49,50]. Both datasets were collected during the growing season (April-September) and were combined for complete aerial coverage of the lower Quinault River to represent summertime conditions when deciduous coverage is at a maximum. Riparian tree and vegetation height were determined from this lidar imagery following standard methods [51]. Vegetation heights were calculated in ArcGIS by subtracting ground returns by maximum height returns. Raster resolution was downsampled to a cell size of 9.0 m^2^ from LiDAR point data (~9 points/ 1.0 m^2^), using the maximum tree height measured. Two raster layers were created, one for vegetation height and one for ground elevation. A river polygon layer was constructed along the river’s low-flow bank width utilizing ortho imagery from the USGS National Map. River channel migration evident from the orthoimage was reconciled with LiDAR data to improve representation of vegetative shading. Changes were small, < 100-meter sections, except where there was evident channel migration along a 3 km section of the river roughly 23 km downstream of the lake outlet. The river channel polygon was edited to estimate the historical channel present during LiDAR acquisition; this was done to more accurately represent and sample river shading from the lidar data and was not thought to substantially differ from shading in the present channel nor heavily influence solar fluxes on the 41 km modeled channel. Finally, a USGS 3DEP 30-m digital evaluation model (DEM) [52], combined with a DEM from LiDAR ground returns, was combined into a raster layer to provide regional topography. This regional DEM was used to measure topographic shading (i.e., mountains and hillsides).

With these elevation raster layers and delineated channel, the TTools routine was run to calculate river elevation, river width, topographic shading angle, and vegetative shading height at each input node. First, the channel aspect and widths were calculated at each node using the river channel polygon. Next, the river elevation and gradient were calculated for each node based on the DEM layer. The topographic shade angle was then calculated in the east, west, and south directions at each node with a search distance of 20 km based on the DEM. This topographic shading at each node was sampled using the "topographic angle tool" from TTools. The topographic angle determination is complicated by distance from the sampling point. TTools identifies the near-field shade angle from the local maxima of the first 25 cells (750 meters for the 30-m DEM) of the DEM and a far-field shade angle from the local maxima out to 20 km. The larger of the two angles is reported for each node’s search direction [40]. Finally, river shading was calculated at each node using the localized vegetation height layers. For each node, seven radial transects, each with ten sampling intervals spaced at ten meters apart, were sampled for vegetation height (see Boyd and Kasper [40] for a detailed explanation).

River geometry was defined by channel bottom width and channel angle (z). In most areas, the river is relatively shallow and wide, resulting in a large width to depth ratio

(40:1–100:1) for all discharge measurement locations on the main channel. The wetted channel width measures from TTools were used as bottom widths for model input (z = 0) since neglecting channel side angle had no appreciable impact on temperature calculations given this large W:D ratio. Other literature-based model inputs are summarized in Table 1 with their source.

**Table 1:** Input values used in Heat Source version 9.

Parameter	Value^1^	Reference^2^
Meteorology		
Relative humidity [%]	0.0–100.0	Measured
Air temperature [°C]	8.3–31.9	Measured
Wind velocity [m s^-1^]	0.0–12.1	Measured
Cloudiness [%]	0.0–87.0	Estimated
Morphology		
Mean side slope ratio [dimensionless]	0.0	Estimated
Channel bottom width [m]	22.7–200.6	Estimated
Deep alluvium temperature [°C]	12.0	Estimated
Thickness of hyporheic/substrate layer [m]	0.12	Calibrated
Hyporheic exchange [%]	0.0	Calibrated
Porosity [unitless]	0.400	Freeze and Cherry [53]
Sediment thermal diffusivity [cm^2^sec^-1^]	1.54	Pelletier [54]
Thermal conductivity of sediment [Wm^-1^°C^-1^]	0.0064	Pelletier [54]
Manning’s roughness coefficient [dimensionless]	0.035	Arcement and Schneider [55]
Tributary Inflows		
Inflow temperature (boundary and tributary) [°C]	9.80–22.70	Measured and Estimated
Inflow discharge (boundary and tributary) [cms]	0.02–71.60	Estimated
Land Cover		
Topographic shade angle [degrees]	0.5–52.5	Estimated
Elevation [meters]	8.5–99.9	Estimated
Tree height [meters]	0.0–82.3	Estimated
Canopy density [%]	75–90	Estimated
Vegetative overhang [meters]	0–2	Estimated

^1^Value range shows min and max for the modeling period.

^2^Measured values are from continuous field sensor data. Estimation refers to values from GIS layers, orthoimages, or calculated based on other models. Detailed descriptions of measured, estimated, and calibrated values are found in the text. Literature constants and their sources are listed. Model inputs as defined in Bond et al. 2015 [18] and Boyd and Kasper 2003 [40].

### Meteorology

Meteorological data for the Heat Source model were retrieved from MesoWest’s database of Remote Automatic Weather Stations (RAWS) data. Weather data for the Quinault River basin are sparse, limited to a single station along the lower reach: Black Knob near Quinault (Station ID: BKBW1). Direct observations of hourly air temperature, wind speed, and humidity from BKBW1 were used as inputs for the model. Cloudiness was not directly measured at the station, so cloudiness was estimated from measures of solar radiation, E_o_. Cloudiness, measured as a proportion of light blocked by clouds (0–1), was estimated based on the ratio of observed solar radiation, E_o_, to modeled maximum radiation, E_m_. E_m_ for the reach was determined by running the Heat Source model (see below) with vegetation height and topographic shading angle set to zero. E_m_ values at the BKBW1 station were divided by these date and time-specific maximums and converted to cloudiness values using a previously established cubic regression model [56]. Specifically, cloudiness was estimated by iteratively solving the equation reported in Luo et al. [56] with our values of E_o_/ E_m_.

### Model calibration

With the inputs generated, river temperature was predicted using Heat Source (v. 9.0.b22), built on Python 2.7. Model input and resulting output files are available via a USGS Sciencebase data release [57] available at https://doi.org/10.5066/P9XGI6GS. The Heat Source model predicted river temperature over the 41-km reach, from RK 54.1 to 13.5, over a spatial resolution of 500 meters for June 10th to September 18th, 2019, at a two-minute timestep and the initial flush condition set to 5 days. Observed data from August 1st, 2018, to September 17th, 2018, except meteorology and temperature data for both the boundary condition and tributary inflow, were used for model validation. Missing tributary (Cook Creek) and a short period of boundary condition water temperature in 2018 were estimated using a random forest regression model, detailed in the supplemental information. The model inputs used, and references to their source, are listed in Table 1.

Additional parameters not previously discussed include setting evaporation methods and deep alluvium temperature. The evaporation rate calculation was set to "Mass transfer" [58] with a wind offset coefficient of 2.21 x 10^−9^ and a slope coefficient of 0.0 [59]. Deep alluvium temperature, which is held constant in the model, was estimated at 12.0°C, which is 1.2°C above the mean annual air temperature [60]. The model was calibrated based on observed data before running vegetative and climate change scenarios.

The thickness of the hyporheic/substrate layer and hyporheic exchange parameters were calibrated following procedures outlined in Bond et al. [18]. Model fit was assessed on both an hourly and daily time step for calibration and validation periods based on three criteria: bias, root mean square error (RMSE), and Nash-Sutcliffe Modeling Efficiency (NSE) value. Bias measures whether, and to what extent, model outputs over or underestimate observed values. RMSE assesses goodness-of-fit to observed values; lower values indicate a better fit [61]. NSE provides a normalized statistic for how modeled variance compares to measured variance; an NSE = 1 is a perfect match to the observed data [61].

Eight candidate models were run (Table S2 in S1 File), and measures of fit were calculated from hourly predicted temperatures at all locations where a temperature sensor was present in 2019 (Fig 1). Candidate models were selected to bracket expected measures of calibrated parameters. Model fit was assessed first for hourly predicted data by comparing observed values at the six main-stem temperature monitoring locations. The two best models were carried forward, and model fit was calculated for daily average temperature values compared to the observed daily average temperature at the same six main stem monitoring locations. Daily model fit, on averaged water temperature, was calculated because it is used as input to the fish bioenergetics model. Since the bioenergetics model (described below) estimates fish growth on a daily timestep, daily averages for the candidate models and measures of fit were calculated at all main-stem monitoring sites. Measures of fit were then averaged across monitoring locations.

### Riparian and climate simulations

**Fig 3:**
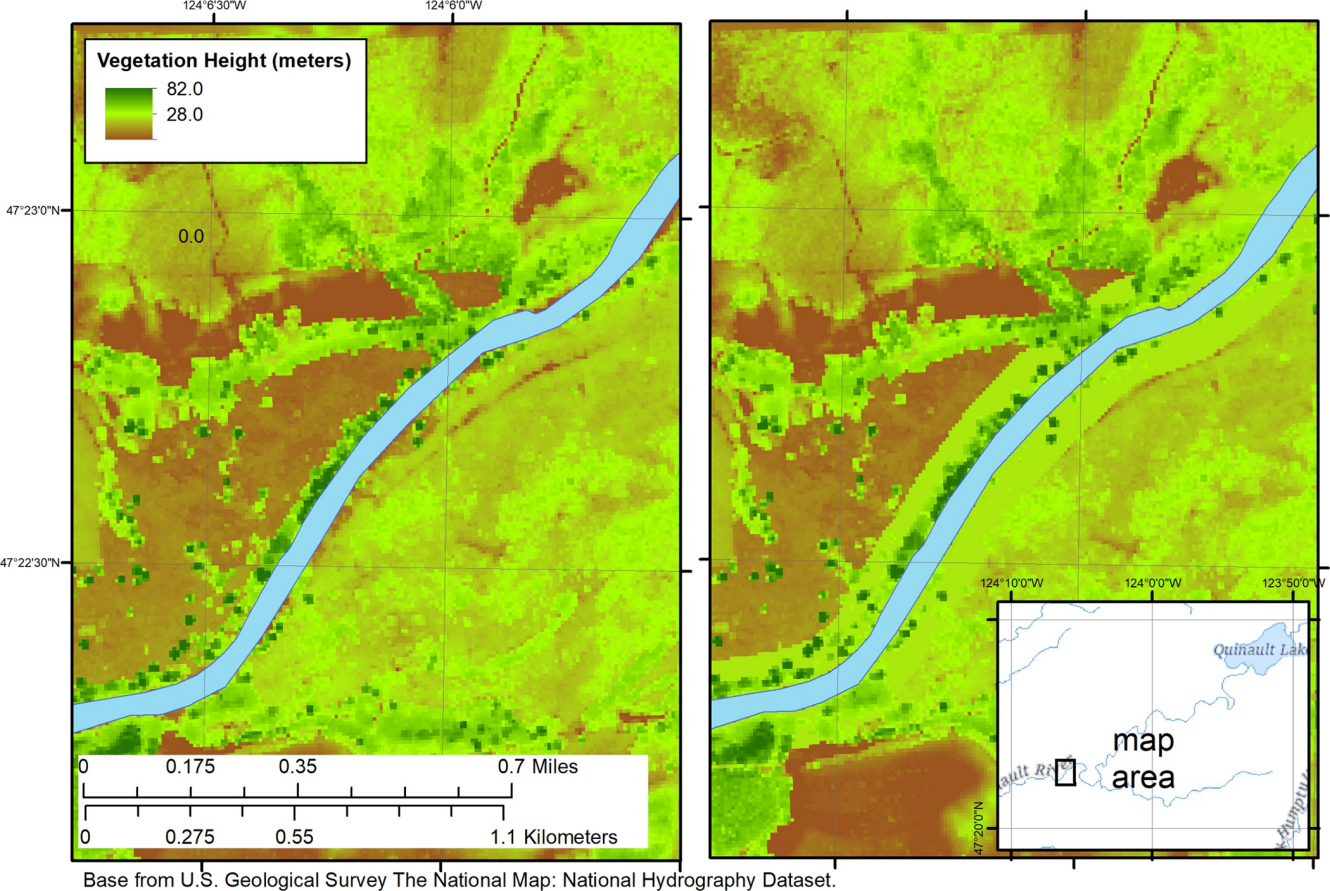
Example of riparian vegetation addition of nearshore river buffer with increased vegetation heights (in meters). The left panel represents current vegetation height, and the right panel represents a minimum 24.4-meter (80 feet) tree height within a 100-meter buffer from the streambank.

We simulated both future reductions and increases in riparian shading by varying vegetation height for model input (Table 2). For riparian vegetation reduction, river temperature was predicted based on removing nearshore vegetation over the entire reach and along both banks; although extreme, the complete removal of vegetation serves two purposes: 1) to present the upper thermal limit of possible outcomes over a long stretch of river, and 2) to help illustrate where water temperatures are most sensitive to vegetative loss. For future riparian vegetation increases, a 100 m buffer was designated in ArcMap (example, Fig 3). Within this buffer, tree heights were set to a minimum of 24.4 m (80 feet), which is on the lower range of tree heights for mature mixed coniferous and deciduous forests [18,62]. The heights of trees in the simulated buffer region that were already taller than 24.4 m were kept at their original height, as growth rates slow once maturity is reached.

**Table 2:** Summary of climate and vegetation scenarios.

Variable	Name	Definition
Climate	2020	Average predicted hourly air temperature during 2010–2029
	2040	Average predicted hourly air temperature during 2030–2049
	2060	Average predicted hourly air temperature during 2050–2069
	2080	Average predicted hourly air temperature during 2070–2089
Vegetation	Increase	300-foot riparian buffer allowed to grow to an early old growth height of 24.4 m (80 ft)
	Current	Current riparian condition
	Loss	Loss of riparian vegetative buffer throughout the lower river
Representative Concentration Pathway (RCP, carbon emission scenarios)	4.5	Medium emissions scenario
	8.5	High emissions scenario

The World Climate Research Programme’s (WCRP’s) Coupled Model Intercomparison Project Phase 5 (CMIP5) multi-model dataset downscaled future climate scenarios for the Quinault River basin at a 1/16-degree scale. Future air temperature data were retrieved from the bias-corrected and downscaled WCRP CMIP5 Climate projects archive (http://gdo-dcp.ucllnl.org/downscaled_cmip3_projections/) using the Localized Constructed Analogs (LOCA) hydrology dataset [63] for the Hadley Centre’s Earth System Model, HadGEM2-ES [64] at a daily time step. The HadGEM2-ES was used for its performance in the Pacific Northwest Region [65]. Data are reported as year-specific and represent daily averaged meteorological predictions for 20-year periods as follows: 2020: 2010–2029, 2040: 2030–2049, 2060: 2050–2069, and 2080: 2070–2089. Two carbon emissions scenarios were used—(RCP 4.5 (medium emissions scenario) and 8.5 (high baseline scenario) [38]. The LOCA CHIMP5 projects temperature at a daily timestep. Diurnal variation in hourly temperature data was simulated by calculating the difference between daily mean temperatures and each hourly measured temperature in 2019. The difference was then added to the daily average predicted by the climate model at an hourly time step [66]. Water temperature boundary conditions, at the outlet of Lake Quinault, were held constant in future climate scenarios (see the discussion for potential limitations).

To summarize temperature data, a 7-day average daily maximum (7DADM) water temperature was calculated for the period, and differences from temperatures modeled from “current" riparian conditions were calculated.

### Fish bioenergetics model

Chinook salmon spatially explicit potential growth was predicted over the summer season utilizing the Wisconsin Bioenergetics model [40] parameterized for Chinook salmon [31,30] at each location with temperature model outputs (every 500 km). Briefly, fish bioenergetics models are energy mass balance equations that predict growth based on the energy available from prey and the energy cost of fish metabolism and waste. This takes the form of Growth = Consumption—Metabolism–Waste. Consumption, metabolism, and waste components are defined by underlying equations that describe their respective energetic cost [40] and predicts growth on a daily time step. All fish bioenergetic model input and output files are available via a USGS Sciencebase data release [54] available at https://doi.org/10.5066/P9XGI6GS.

Bioenergetics models that simulated potential growth used inputs of prey and predator (i.e., juvenile Chinook salmon) energy density, thermal regime, prey (i.e., diet) proportions, percent indigestible prey, starting predator weight, and percent consumption. The thermal regime for the modeling period was from Heat Source outputs. All other inputs were derived from literature values as follows. Predator and prey energy content, diet proportions, indigestible prey, and consumption rates were held constant for the modeling period (Table 3). A starting weight of five grams was chosen to represent juveniles roughly 3–5 months post-hatch and is within the range empirically determined from other studies that have measured the weight of age-0 Chinook salmon [67,68]. A predator energy density of 4.7 kJ/g was selected based on a study of freshwater juvenile Chinook salmon [69], and an average prey energy density of 3.0 kJ/g was selected, representing low-energy diets (i.e., aquatic insect dominant) typical of juvenile salmonids in warm-water streams and rivers in mixed coniferous/deciduous forests [8,70]. These input values represent a reasonable estimation for modeling and comparison of temperature effects on growth. Temperature outputs from temperature modeling scenarios were formatted for Fish Bioenergetics 4.0 using a custom R script. This script produced daily averages of river temperature at each modeling location (i.e., every 500 m).

**Table 3:** Bioenergetic model inputs for juvenile Chinook salmon growth scenarios.

Input	Value
Starting weight [g]	5.0
Model run dates	June 10th- September 18th, 2019
Consumption level [%Cmax]	50
Predator energy density [KJ/g]	4.7
Prey energy density [KJ/g]	3.0
Temperature [°C]	Variable based on scenario

We used Fish Bioenergetics 4.0 [71], an R-based implementation of the Wisconsin Bioenergetics model, to model juvenile Chinook salmon growth in the lower Quinault River from June 10th, 2019, to September 18th, 2019, corresponding to river temperature modeling periods. Juvenile were given an estimated feeding rate (%Cmax) of 50%. Cmax is defined as the proportion of a theoretical maximum consumption amount. This theoretical maximum is calculated by the model on a daily timestep based on their wet weight and is expressed here as a percentage. The average consumption rate of 50% is within the range of values reported for salmonids in recent studies [26,27] and the impact of different consumption rates on growth is considered in the discussion section. Bioenergetics models were batch run at each node (n = 82) across the entire reach to predict daily growth at each predicted river temperature node allowing for longitudinal visualization of potential growth at different river temperatures along the entire modeled reach. Potential growth is reported in grams per day. Statistical differences of Chinook salmon growth amounts were compared among scenarios. Normality was checked using a QQ plot, and statistical differences were calculated using a parametric factorial analysis of variance with interaction (α = 0.05).

In addition to juvenile potential growth, we compared predicted river water temperature to life history-specific temperature thresholds and general mortality thresholds for all life-stages and salmonid species by calculating the percent of time a section of the river exceed the threshold for each scenario [11].

## Results

### Field measurements

Water temperature records for 2019 were measured at all locations from June 10th until September 18th, 2019. A NIST-verified thermistor independently verified HOBO temperature loggers during field visit readings. Temperature measurements in cross-sections where temperature loggers were deployed were ±0.2°C of each other in all cases, indicating that measured temperature was representative of a well-mixed channel and that thermistors were not placed in anomalously cold or warm areas of the channel. Water temperature records were discontinuous in 2018, so only summer 2019 was predicted for use in bioenergetics modeling. The mean measured hourly temperature for all sites was 18.9°C, with a maximum of 23.6°C and a minimum of 14.3°C. Daily-mean and study-mean temperatures generally decreased moving downstream, indicating cooling of lake outlet temperatures throughout the lower reach during the study period.

The measured tributary flow was low, less than 2% of main-stem flow at five locations in the lower reach. Cook Creek (USGS Station No. 1203951185, Table S1 in S1 File) and Boulder Creek (USGS Station No. 1203950980, Table S1 in S1 File) had flows above 2% of main-stem discharge (7.9% and 2.4%, respectively). Due to the difficulty accessing sites, discharge measurements were not repeated in 2019. Percent of main flow calculations were used in 2019 since summer flow conditions were similar between 2018 and 2019 with a median daily average flow of 18.83 m^3^/s in 2018 and 16.14 m^3^/s in 2019 over the modeling period of June 10^th^ to September 18^th^ at the upper gage (NWIS, https://waterdata.usgs.gov/nwis) [47]. Estimated tributary flow accounted for an average of 69.5% of the difference in daily mean flow between the upper and lower gage (time of travel corrected mean discharge is 20% higher at the lower gage) during 2019. Estimated tributary contributions were as low as 26% of the difference in daily mean flow between the upper and lower gage during the lowest flow periods of the summer and during precipitation events.

### Water temperature modeling

**Fig 4:**
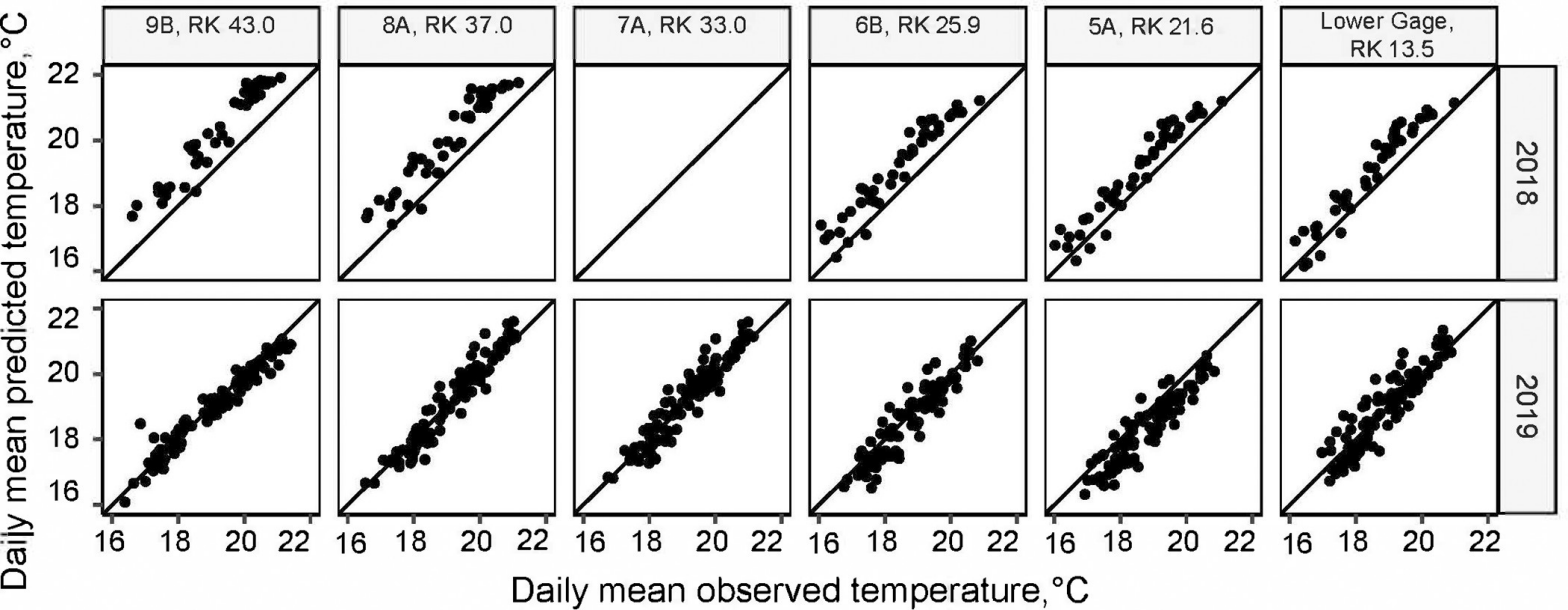
Heat Source model fit. Hourly predicted versus observed temperature values for the final Heat Source model at all monitored temperature locations in 2019 (n = 6) and 2018 (n = 5).

Both spatially averaged hourly, denoted by “.h”, models 5.h and 7.h performed well based on fit estimates (Table S2 in S1 File). Model 7.h performed best when evaluating based on predictions of daily averages (Table S3 in S1 File) with the lowest measures of bias and RMSE and an NSE closest to one. To assess spatial model fit of the final model, prediction at each river node where observed temperatures were available were calculated and have a similar error (Table 4 and Fig 4).

**Table 4:** Measures of calibrated water temperature daily model fit by continuous monitoring location.

USGS NWIS Station name	USGS NWIS Station no.	Latitude	Longitude	Bias (°C)	RMSE (°C)	NSE
QUINAULT R BLW PRAIRIE CR (9B) NR QUINAULT, WA	12039504	47.430867	-123.949381	-0.09	0.33	0.93
QUINAULT RIVER (8A) NEAR NEILTON, WA	12039507	47.422602	-124.003967	-0.05	0.36	0.97
QUINAULT R BLW TEN O’CLOCK CR (7A) NR NEILTON, WA	12039509	47.401431	-124.034670	-0.07	0.38	0.97
QUINAULT RIVER BLW COOK CR (6B) NR NEILTON, WA	12039511	47.371117	-124.082879	0.13	0.41	0.96
QUINAULT RIVER BLW JOE CREEK (5A) NR NEILTON, WA	12039514	47.372716	-124.107716	0.44	0.58	0.92
QUINAULT RIVER NEAR THOLAH, WA	1203951610	47.357777	-124.184444	-0.05	0.46	0.95

**Abbreviations:** RMSE = Root Mean Square Error (RMSE); NSE = Nash-Sutcliffe Modeling Efficiency.

Validation of model 7.d with river temperature from 2018 showed a good fit (Table S3 in S1 File and Fig 4) compared to observed temperatures indicated by the low RMSE values at each monitoring location, but the predicted temperatures in 2018 were biased higher (0.82°C) than observed to a greater extent than in 2019 (0.04°C). This bias was likely the result of higher temperatures measured at the upper boundary due to a temperature sensor that was mostly in a shallow area near the upstream gage. This sensor was moved to a new location on September 5th, 2018. Generally, the validation confirmed the fit of the model.

### Modeled stream temperature scenarios

**Fig 5:**
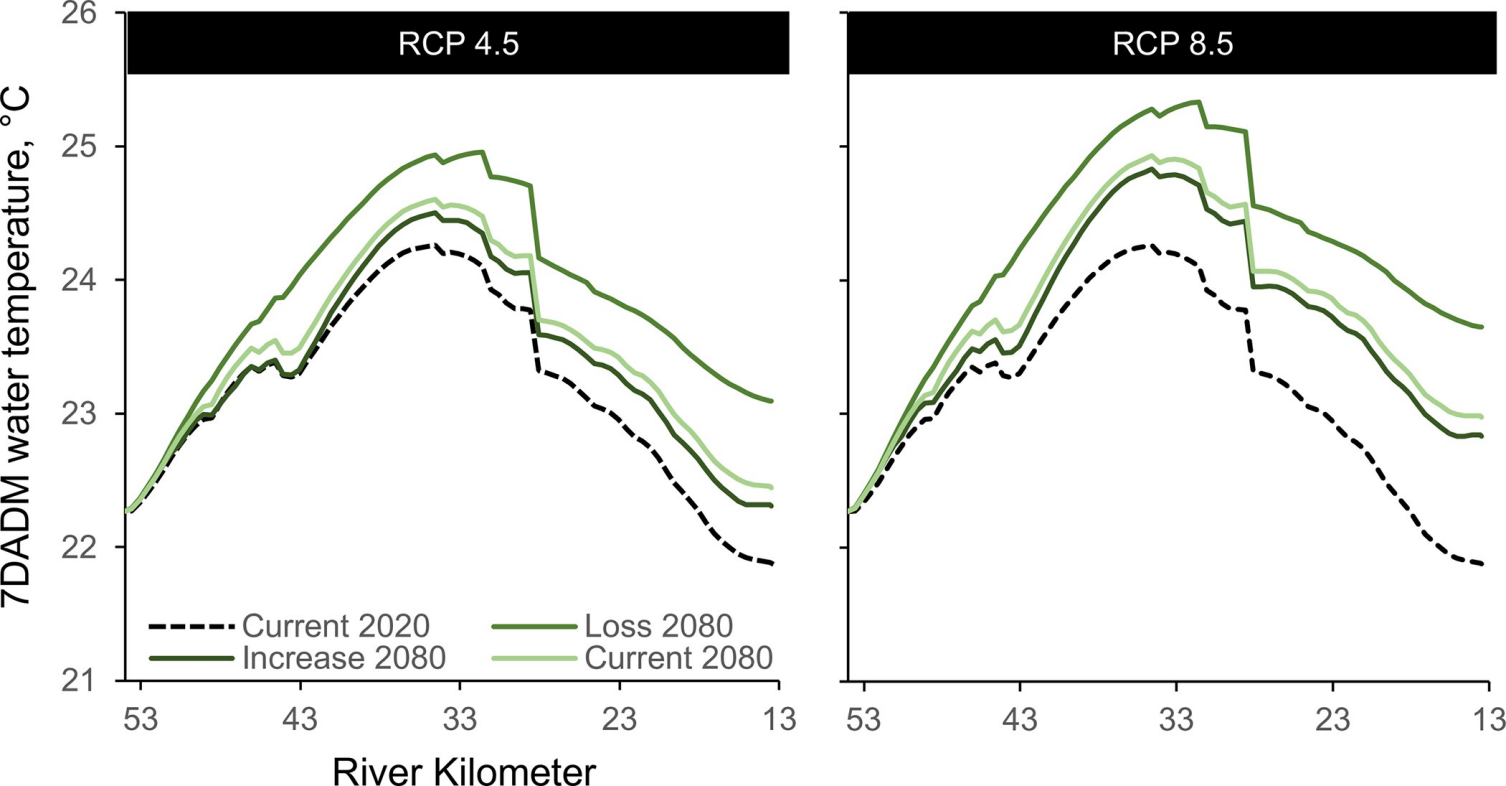
Maximum 7DADM water temperature. Water temperature predicted at each node under two future climate emission scenarios, RCP 4.5 and 8.5, and three riparian vegetation scenarios, as compared to current conditions.

Model output predicted increases in river temperatures from loss of vegetation and increases in air temperature under future climates, while increases in riparian shading from increased vegetation height lowered current temperatures (Fig 5). Air temperature increased substantially under the RCP 4.5 and 8.5 emission scenario as predicted by the GCM in the Quinault River basin. By 2080, air temperature was projected to increase by an average of 2.11°C (RCP 4.5, SD ± 0.63°C) to 3.48°C (RCP 8.5, SD ± 0.59°C). These increases in air temperature resulted in increased predicted water temperature. By 2080, 7DADM water temperatures in the lower Quinault River increased by 0.17 to 0.70°C under RCP 4.5, or 0.44 to 1.00°C under RCP 8.5. Increases in vegetation height and riparian buffers substantially reduced the amount of this increase under both RCP emission scenarios (Fig 5). For example, the maximum 7DADM for a specific location in the river increases by as much as 1.78°C by 2080 with the removal of vegetation but only by a maximum of 0.96°C by 2080 (RCP 8.5) with an increase in riparian vegetation. The modeled temperature increase was both spatially and temporally variable. Under current climate conditions, the 7DADM with riparian vegetation removal averaged 0.39°C (SD ± 0.16°C) higher than under current riparian conditions and 0.52°C (SD ± 0.20°C) higher than is predicted with an increase in riparian vegetation height when averaged across the entire reach. Temperature increases were greatest from below RK 49.8, and daily maximum temperatures were greatest near RK 41.8 and near the lower gage, RK 13.5 (Fig 5).

### Water temperature impacts to fish growth

**Fig 6:**
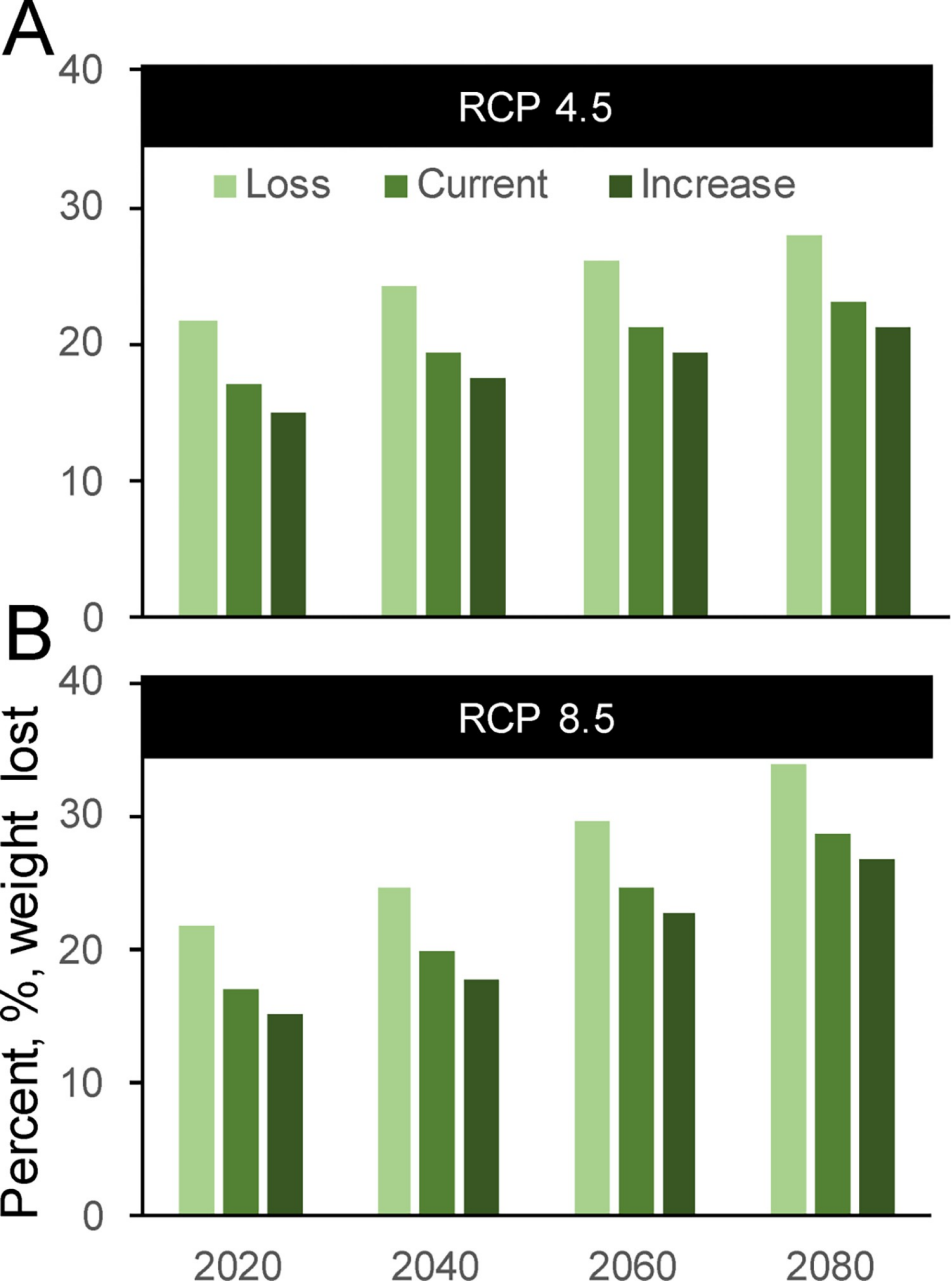
The average percent of weight loss predicted in a 5.0 g juvenile Chinook salmon. Predictions of weight loss are during the summer season in the lower Quinault River under two future climate emission scenarios, RCP 4.5 and 8.5, and three riparian vegetation scenarios compared to the current temperature.

**Fig 7:**
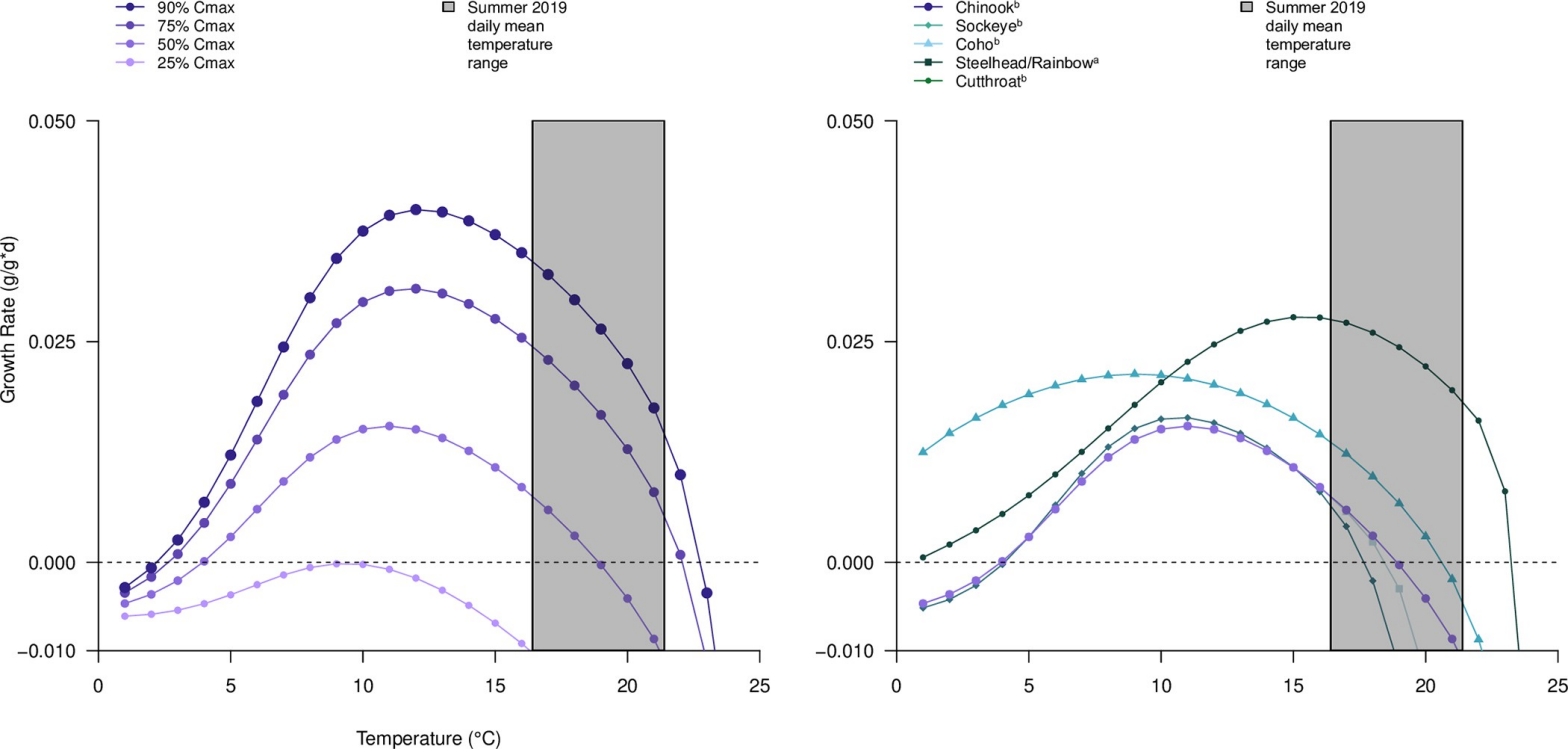
Modeled growth curve for juvenile Pacific salmon and trout. Growth curves are predicted for A) a five-gram juvenile Chinook salmon eating a low energy-dense diet of 3.0 KJ/g; and B) among salmonid species at 50% Cmax. Cmax is the percentage of theoretical maximum amount of prey a fish can eat given their body size (e.g., a 5.0 g fish can theoretically eat up.4 g of food a day, at a Cmax of 50% they consume 0.2 g). Species specific bioenergetic parameters as published in ^a^ [41] and/or ^b^ [71].

Juvenile Chinook salmon potential growth rate, measured in grams/gram*day, was negative over the 101-day modeling period for all scenarios (Fig 6). This result indicates that daily average river temperatures in the lower Quinault River are more frequently above temperatures required for fish growth (i.e., fish are losing mass at these temperatures) (Fig 7A). These results are similar for all major Pacific salmon and trout species potentially present in the lower Quinault River during the summer, except for steelhead and cutthroat trout that have higher thermal tolerances (Fig 7B). Under current conditions, juvenile Chinook salmon with a feeding rate of 50%, lost an average of 0.86 g (SD ± 0.22 g); this loss varied by location. Increasing vegetation height within the basin had a small but significant (p<0.001) improvement of growth throughout the reach, reducing fish mass declines to a mean change of -0.77 g (SD ± 0.26 g). Changes in the thermal regime from riparian vegetation removal resulted in significantly (p<0.001) more weight loss with a mean change of -1.09 g (SD ± 0.15 g). Simulations with an increase in temperature under future air temperature increases predicted significantly greater weight loss (p< 0.001) under all riparian conditions (Fig 6). Interactions were not significant (two-way ANOVA), indicating that differences among riparian conditions did not have a greater effect on fish growth with increases in air temperatures for either a vegetation height increase (p = 0.99) or vegetation removal (p = 0.71).

**Fig 8:**
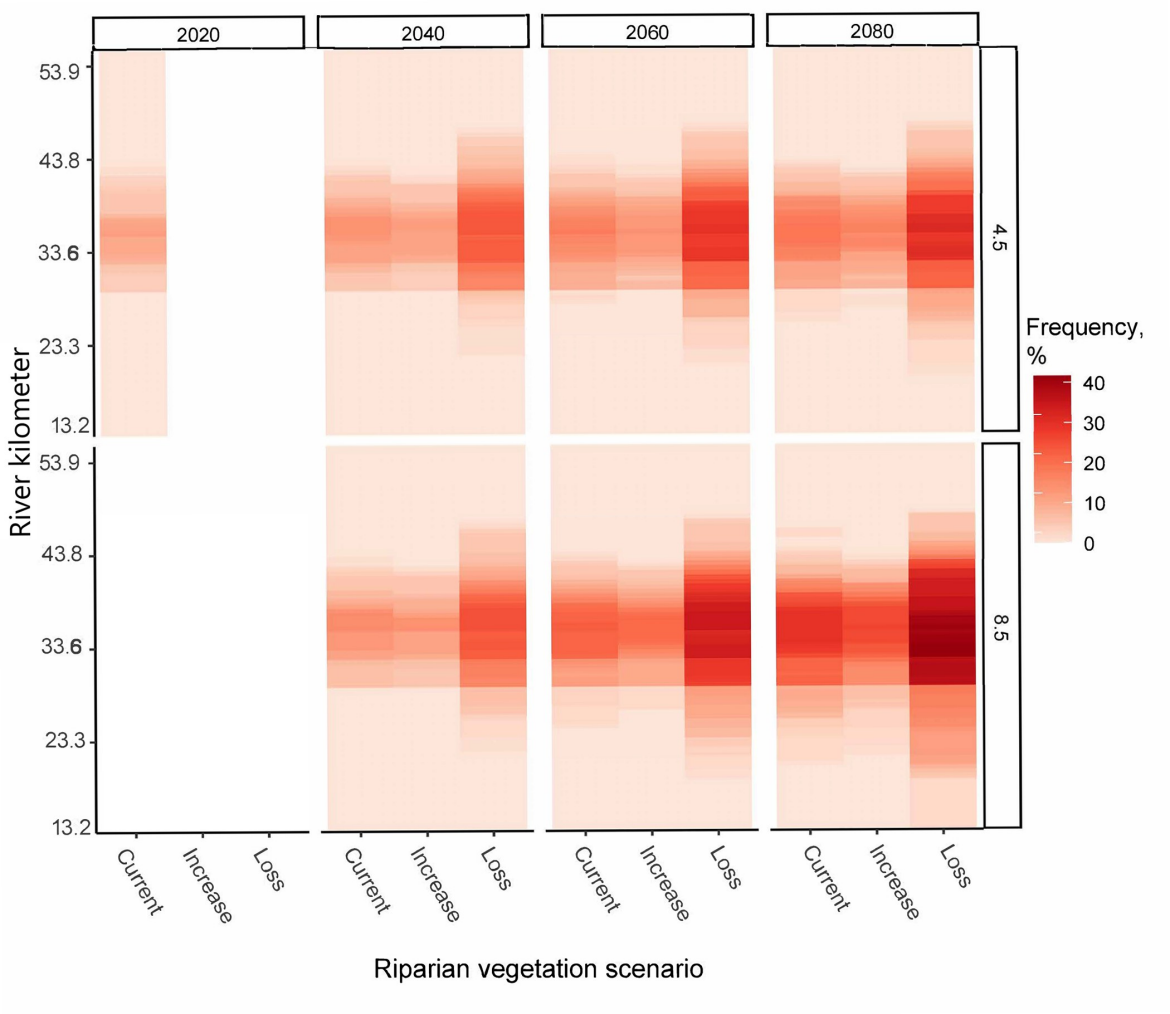
Frequency of predicted lethal water temperature exceedances. Exceedances predicted for adult and juvenile Pacific salmon (non-species specific) [11], in the lower Quinault River (RK 13.2 to 53.9) predicted under future carbon emission projections, RCP 4.5 and 8.5, and riparian shading scenarios during the summer season using a Heat Source water temperature model. Frequency is calculated as the percentage, %, of days with a maximum temperature greater than 24°C at a specific kilometer.

Predicted water temperatures during the summer in the lower Quinault River exceeded temperature thresholds for sensitive salmonid life stages, including smoltification (12–15°C), embryo development (12–15°C), spawning migration (19–23°C), and adult/juvenile mortality (24–26°C) [11]. These exceedances were temporally and spatially variable throughout the lower watershed. Warming climates resulted in an increase in these exceeded temperatures’ spatial extent and frequency (Fig 8). Riparian vegetation retention or increases in riparian shading is predicted to partially mitigate both the spatial extent and frequency (Fig 8). Spatially, high temperatures are concentrated mid-way down the modeled reach. The river mid-stream aspect is oriented in northeast to southwest direction with less topographic and vegetative shading. Thus, temperatures exceeding these thresholds are not homogeneous throughout the lower river (Figs 5 and 8).

## Discussion

Results from linked water temperature and fish bioenergetic growth models of the lower Quinault River, WA, USA demonstrated the ability of riparian shading to protect thermal habitats for Pacific Salmon and trout growth under current and future climate projections. Our modeling predicted that reductions in riparian shading would result in a warmer thermal habitat for juvenile Pacific Salmon and trout, specifically Chinook salmon, increasing energetic costs and reducing fish growth potential during summer months. These impacts would be more severe under projected climate shifts in the future with predicted water temperatures (7DADM) increasing by a maximum of 1.78°C by 2080, with riparian losses under a high carbon emissions scenario, RCP 8.5. We predict that current conditions in the river result in negative summer growth potential for juvenile Chinook salmon and that maintaining or increasing riparian shading protects thermal conditions for rearing juvenile fish from the most severe temperature increases. Additionally, we showed that days with temperatures above salmon mortality thresholds would increase in frequency and duration under future climate scenarios, presenting a risk to all salmon species at all life stages using the river for spawning, migration, or rearing during the summer months. These predicted temperature increases that occur earlier and later into the summer present additional life-stage specific risks to fish. Using these linked models provides natural resource managers developing climate adaptation plans a powerful tool to prioritize spatially explicit mitigation efforts by protecting or increasing riparian shading in areas with the most impact at reducing temperature increases.

### Lower Quinault River thermal regime

The lower Quinault River Heat Source model accurately predicted water temperature along the 41-km reach between the Lake Quinault outlet to the downstream gage (USGS Site no. 1203951610) (Table 4). As expected, increases in air temperature predicted from regional climate models increased water temperatures. Observed summer air temperatures during our study were only slightly higher than seasonal averages, in 2018 (+0.2°C) and 2019 (+0.4°C), calculated since 1990 for the western coast of Washington [72], suggesting that the modeling period was representative of a near-average summer for the region and was an appropriate period to compare to future increases. Like other studies [18,73], we predict increases in river temperature under future climate projections. Increases in water temperature estimated by Heat Source models of the lower Quinault River following riparian vegetation removal and increased air temperature were consistent with results from statistical [74,75] and process-based models [76,77] of other rivers within the Pacific Northwest. Heat Source models of the lower Quinault River showed that increased riparian tree height modestly reduced 7DADM (-0.13 ⁰ C) compared to current riparian vegetation conditions; modeled loss of riparian vegetation resulted in an increased 7DADM (+0.39 ⁰ C), illustrating the importance of maintaining riparian buffers along the river to reduce thermal loading of the river.

The Quinault River is unique among other major rivers on the Washington coast due to the presence of a sizeable, relatively shallow moraine-dammed lake mid-point along its course. The lake’s large-exposed surface area receives substantially more solar radiation than the river flowing into the lake, resulting in increased temperatures. In this way, the lake functions much like artificially impounded systems that increase downstream temperatures and reduces the magnitude of diurnal temperature periodicity [78,79]. Additionally, the lake’s presence indirectly impacts temperature due to reduced downstream loading of large woody debris [39] that would otherwise have the potential to create additional thermal refuge locations in the lower river. With future increases in air temperature, the lake’s influence on the lower river’s temperatures will likely increase. This increase in downstream temperatures is predicted in artificially impounded streams and rivers [80,81]. Yet, in the Quinault River, downstream temperatures cannot be mitigated by controlled deep hypolimnion releases. The resulting temperature increases on the Quinault River indicate that low-elevation river temperatures, that are moderated by moraine-dammed lakes [82], are likely to be sensitive to increasing climate temperatures. Fish species whose life histories use and, in some cases, have benefited from these systems [83], such as salmonids, will face additional pressure from changing thermal regimes necessitating considerations and research into appropriate fisheries management and conservation strategies.

### Model uncertainty

All models are simplified representations of reality, and our results have inherent uncertainty. Reported RMSE based on mean hourly temperature (1.07°C) was slightly higher than a heat source model used on a 1 km reach of the NF Salmon River [18], but similar to studies that modeled larger (10 km to 437 km) river reaches [84,85]. Model performance, assessed by mean daily temperature (RMSE <0.5°C), was improved relative to mean hourly temperature. Errors in hourly water temperature were less critical as input to the bioenergetics model, which requires mean daily water temperature. Uncertainty about the role of hyporheic exchange [18,86], groundwater inputs, and meteorological conditions coupled with the 41-km study-reach length likely contribute to Heat Source modeling error. It was not feasible to measure hyporheic exchange on a spatially explicit basis, and thus it was a calibrated variable and held constant throughout the entire reach. Hyporheic exchange influences stream temperature [87] and the use of a constant value likely introduced additional error in our model, though with likely less impact to daily mean temperatures [87] used for bioenergetics modeling. Microclimates created by riparian vegetation were not well characterized by our study, which would have necessitated a network of meteorological stations throughout the basin to better characterize meteorological inputs throughout the full 41 km reach. Microclimate changes attributable to riparian vegetation include reductions in wind speed and air temperature and increases in humidity [88,89]. Still, reduced solar radiation from shading is the predominant mechanism by which riparian vegetation affects river temperatures [21].

The predictions of future climate changes have their own limitations. Our analysis didn’t investigate changes in the timing and quality of snowpack, flow in the upper river, and related increases in groundwater temperature likely under future climates [90–93]. Additionally, changes in Lake Quinault’s temperature were not modeled, and we neglected the concurrent increase in the lake temperature at the river boundary, which will likely compound water temperature increases estimated from riparian vegetation removal and projected air-temperature increases. Because of these limitations, water temperature increases in the river could be higher than what is predicted here. The results from our modeling are consequently conservative estimates of higher temperatures in the basin.

### Salmonids in a warm and warming river

The warm water temperatures observed and modeled in the lower Quinault River negatively impact juvenile Chinook salmon growth potential under current and future climates. Growth modeling of other anadromous salmonid species would show nearly identical results, as evident by modeled growth curves for other Pacific salmon and trout species (Fig 7B), though juvenile cutthroat and steelhead trout would likely experience positive growth under current conditions due to higher thermal tolerances (Fig 7B). Under our bioenergetics scenarios, juvenile Chinook salmon experience negative growth under current conditions, even with increased riparian shading from increased vegetation. Given these stressed conditions, it’s likely that fish are already avoiding the main stem as primary rearing habitat, either by earlier migration to the lower estuary [94], upstream migration [95], or by seeking daytime refuge in tributaries and cold-water pools [96–98]. Juvenile fish may be able to avoid these warm temperatures, particularly during August, when temperatures are highest and predicted to have the most considerable increase in the frequency of thermal threshold exceedances. Under future climates, the extent and duration of these high-temperature periods increased, reducing suitable habitat throughout more of the river.

Despite these high temperatures, feeding rates, assuming adequate forage, may compensate for some of this lost growth. Beauchamp [8] discusses how diet and consumption rate can compensate for fish under significant thermal stress. Our modeling indicates that positive growth can be achieved at a daily mean temperature of up to 22.7°C if fish can feed at 90% of their maximum consumption rate versus 18.9°C at 50% of their maximum consumption rate (Fig 7). Given adequate prey availability, the positive growth rates of salmon predicted at high water temperatures are confirmed by recent laboratory studies [99]. Additional gains in growth are possible with improvements in diet quality. Increases in riparian vegetation can increase inputs of high-energy prey items, such as terrestrial insects, that would also improve potential juvenile salmon growth in high-temperature rivers and streams [100]. These terrestrial prey subsidies are shown to comprise up to half of the salmonid diets [100] with higher subsidies of terrestrial prey in rivers with deciduous versus coniferous riparian vegetation. To improve our understanding of potential growth in the lower Quinault River, additional data on salmonid diet conditions, consumption estimates, and terrestrial and aquatic prey abundance are needed. Still, it’s clear from our modeling results that fish are under significant metabolic stress at current and predicted summer temperature levels. The relationship among riparian shading, river temperature, and potential fish growth would remain under different diet conditions.

High water temperatures are not only a concern for rearing juveniles but also salmonids at all life-stages in the river. Our results predict daytime maximum temperatures that are lethal to salmon at all life stages during parts of the summer and higher than thresholds for migration, embryo development, and smoltification during nearly every summer day. Of particular concern is that the timing of these threshold exceedances extends longer into the fall season, which could create a thermal barrier to migrating fish.

Salmonids use heterogeneous temperatures in both lentic and lotic systems in the Pacific Northwest [101,102]. Throughout the lower Quinault River, the use of a one-dimensional temperature model here neglects consideration of potentially important cold-water refuge resulting from groundwater inputs and shaded deep pockets created from the presence of large woody debris. Protection of these cold-water refuge may 1) be necessary for sustaining the populations observed today (and potentially more so in the future) and 2) benefit from developing a more complex model and input data that can specifically address these locations. Additionally, further work to assess how these high river temperatures affect water temperatures in the lower estuary would support management decisions. The estuary temperature could significantly impact ocean-type juveniles that migrate early and spend more time rearing in the estuary.

## Conclusions

Although current temperature conditions suggest the Quinault River of Washington State is well shaded, evidenced by the limited temperature mediation under increased riparian vegetation, current summer conditions offer low growth potential for juvenile salmon and trout. Measured temperatures in the river were near or above physiological thresholds and could present a barrier to migrating spawners [103,104]. Given projections of increased air temperature, riparian vegetation in the lower Quinault River is important for limiting future water temperature increases. Further study is warranted into the fine-scale presence of cold water refuge in the lower river that may provide additional cooler habitats not captured in this model and confer the most critical restoration and protection opportunities. The spatially and temporally explicit predictions of river temperatures, fish growth potential, and fish physiological exceedances provide vital information to natural resource managers weighing the benefit and extent of mitigation efforts; these techniques are well suited to the management of other systems. This study shows the importance of physical shading for salmonids in rivers with high temperatures relative to thermal tolerances, influenced by the presence of a large, naturally impounded lake.

## Supporting information

S1 File Supporting figures and tables. This PDF file contains (1) S1 Table. (2) 2018 missing temperature record estimation (3) S1 Fig. (4) S2 Table. (3) S3 Table.
(DOCX)
