# Supplementary material for: Assessing climate change impacts on Pacific salmon and trout using bioenergetics and spatiotemporal explicit river temperature predictions under varying riparian conditions
Source: PLoS One. 2022 May 20;17(5):e0266871. doi: 10.1371/journal.pone.0266871 (PMC9122258; doi:10.1371/journal.pone.0266871)
Supplement: S1 File — This PDF file contains (1) S1 Table. (2) 2018 missing temperature record estimation (3) S1 Fig. (4) S2 Table. (3) S3 Table. (DOCX) [file pone.0266871.s001.docx]

**Supporting Information**

**Supporting Information for: “Assessing climate change impacts on Pacific salmon and trout using bioenergetics and spatiotemporal explicit river temperature predictions under varying riparian conditions”:**

**Table S1.** Locations of monitored and estimated temperature for use in the construction of a Quinault River temperature model. Period of temperature records and calculated percent (%) of gaged flow (tributaries only). [U.S. Geological Survey (USGS); National Water Information System (U.S. Geological Survey 2021, NWIS. [47])]

| **USGS NWIS Station name** | **USGS NWIS Station no.** | **Latitude** | **Longitude** | **Reach** | **% of gaged flow** | **Period of temperature record** |
| --- | --- | --- | --- | --- | --- | --- |
| QUINAULT RIVER AT QUINAULT LAKE, WA | 12039500 | 47.457633 | -123.889375 | Main | -- | 8/1/2018 to 9/17/2018^1^  6/10/2019 to 9/18/2019 |
| QUINAULT R BLW PRAIRIE CR (9B) NR QUINAULT, WA | 12039504 | 47.430867 | -123.949381 | Main | -- | 8/1/2018 to 9/17/2018  6/10/2019 to 9/18/2019 |
| QUINAULT RIVER (8A) NEAR NEILTON, WA | 12039507 | 47.422602 | -124.003967 | Main | -- | 8/1/2018 to 9/17/2018  6/10/2019 to 9/18/2019 |
| QUINAULT R BLW TEN O'CLOCK CR (7A) NR NEILTON, WA | 12039509 | 47.401431 | -124.03467 | Main | -- | 8/1/2018 to 9/17/2018  6/10/2019 to 9/18/2019 |
| QUINAULT RIVER BLW COOK CR (6B) NR NEILTON, WA | 12039511 | 47.371117 | -124.082879 | Main | -- | 8/1/2018 to 9/17/2018  6/10/2019 to 9/18/2019 |
| QUINAULT RIVER BLW JOE CREEK (5A) NR NEILTON, WA | 12039514 | 47.372716 | -124.107716 | Main | -- | 8/1/2018 to 9/17/2018  6/10/2019 to 9/18/2019 |
| QUINAULT RIVER NEAR TAHOLAH, WA | 1203951610 | 47.357777 | 124.184444 | Main | -- | 2017-12-09 to Present |
| BOULDER CREEK NEAR MOUTH NEAR AMANDA PARK, WA | 1203950350 | 47.436611 | -123.921286 | Tributary | 0.8% | No sensor 2018^2^  6/10/2019 to 9/18/2019 |
| PRAIRIE CREEK NEAR MOUTH NEAR QUINAULT, WA | 1203950370 | 47.436029 | -123.940077 | Tributary | 1.3% | 8/1/2018 to 9/17/2018  6/10/2019 to 9/18/2019 |
| TEN O'CLOCK CREEK NEAR MOUTH NEAR QUINAULT, WA | 1203950790 | 47.409183 | -124.033724 | Tributary | 0.6% | 8/1/2018 to 9/17/2018  6/10/2019 to 9/18/2019 |
| BOULDER CREEK NEAR MOUTH NEAR TAHOLAH, WA | 1203950980 | 47.392679 | -124.050839 | Tributary | 2.4% | 8/1/2018 to 9/17/2018^2^  6/10/2019 to 9/18/2019 |
| COOK CREEK NEAR MOUTH NEAR TAHOLAH, WA | 1203951185 | 47.369300 | -124.061982 | Tributary | 7.9% | 8/1/2018 to 9/17/2018^1^  6/10/2019 to 9/18/2019 |
| JOE CREEK NEAR TAHOLAH, WA | 1203951275 | 47.384027 | -124.079581 | Tributary | 0.5% | 8/1/2018 to 9/17/2018  6/10/2019 to 9/18/2019 |

^1^Records with a portion of period estimated using random forest regression: 12039500: 9/1/2018 to 9/5/2018; 1203951185: 8/1/2018 to 8/30/2018

^2^2018 temperature record for model input from 8/1/2018 to 9/17/2018 was estimated as being equal to nearby Cook Creek base on agreement with the 2019 record.

**Methods to estimate missing temperature records in 2018**

In 2018, three periods of record were missing from temperature monitors used for input into the Heat Source model validation period (Table 1). Two periods were estimated using a random forest regression model; these were USGS site no. 12039500: 9/1/2018 to 9/5/2018 and USGS site no. 1203951185: 8/1/2018 to 8/30/2018. The random forest regression model was constructed from observed data for each tributary used to predict temperature for missing dates during these missing periods. The random forest regression was built using the r package 'randomForest: Breiman and Cutler's Random Forests for Classification and Regression' (Liaw and Wiener 2012). A model for each stream was built using date, hour, air temperature, and observed reservoir outlet temperature. The models produced a good fit. For site no. 12039500 97.2% of the variation was explained, and for Site no. 1203951185 89.8% of the temperature variation was explained by the model. The function 'predict' for the r-package "stat" was then used to predict temperature values for missing data using these constructed models. The entire 2018 period of record was missing for 1203950350 due to a lost sensor. The temperature for site no. 1203951185 was used as input for the model during the 2018 validation period. This estimate was thought to be justified based on the agreement between the two locations in 2019 (supplemental Figure 1).

**Figure S1.** 2019 comparison of measured river temperatures for Site no. 1203950350 and Site no. 1203951185. Line represents 1-to-1 agreement.

A. Liaw and M. Wiener (2002). Classification and Regression by randomForest. R News 2(3), 18-22.

**Table S2.** Measures of Heat Source model fit for hourly (".h") temperature models averaged over six monitoring locations. Bias, Nash-Sutcliffe Modeling Efficiency (NSE), and Root Mean Square Error (RMSE). Candidate models for daily temperature values denoted by (*). NA values represent models that could not converge.

| **Model No.** | **1.h** | **2.h** | **3.h** | **4.h** | ***5.h** | **6.h** | ***7.h** | **8.h** |
| --- | --- | --- | --- | --- | --- | --- | --- | --- |
| Hyp. layer [m] | 0.10 | 0.10 | 0.10 | 0.20 | 0.20 | 0.20 | 0.12 | 0.12 |
| Hyporheic exchange [%] | 0. 0 | 0.1 | 0.5 | 0.0 | 0.1 | 0.5 | 0.0 | 0.1 |
| Bias | -0.26 | -1.54 | NA | 0.76 | 0.55 | NA | 0.05 | -0.74 |
| NSE | 0.44 | -3.99 | NA | 0.19 | 0.53 | NA | 0.49 | -0.90 |
| RMSE | 1.12 | 3.34 | NA | 1.31 | 1.00 | NA | 1.07 | 2.06 |

**Table S3.** Measures of Heat Source model fit for daily average temperature compared to observed daily averages at six monitoring locations, column heading denotes candidate model and 2018 model validation with 7.d. Measures of fit averaged over five measured temperature locations along the 41km reach.

| **Measure** | **5.d** | **7.d** | **2018 Validation** |
| --- | --- | --- | --- |
| Bias | 0.55 | 0.05 | 0.82 |
| NSE | 0.87 | 0.95 | 0.93 |
| RMSE | 0.72 | 0.43 | 0.93 |
